# Cognitive effort devaluation and the salience network: a computational model of amotivation in depression

**DOI:** 10.3389/fpsyt.2025.1581802

**Published:** 2025-09-01

**Authors:** Il Ho Park, Chae Eun Lee, Kyungun Jhung

**Affiliations:** ^1^ Department of Psychiatry and Behavioral Neurosciences, International St. Mary’s Hospital, Catholic Kwandong University College of Medicine, Incheon, Republic of Korea; ^2^ Catholic Kwandong University Industry Cooperation Foundation, Incheon, Republic of Korea

**Keywords:** depression, motivation, apathy, reward valuation, working memory, insular cortex, anterior cingulate cortex, effort discounting

## Abstract

**Introduction:**

Amotivation in depression is linked to impaired reinforcement learning and effort expenditure via the dopaminergic reward pathway. To understand its computational and neural basis, we modeled incentive, temporal and cognitive burden effects, identifying key components and brain networks of cost-benefit valuation.

**Methods:**

Data from 43 psychotropic-free individuals (31 non- or minimally depressed individuals), including Beck Depression Inventory (BDI), Apathy Evaluation Scale (AES), n-back task performance, and resting-state fMRI, were analyzed. Cost-benefit valuation was modeled using loss aversion, learning, temporal, and cognitive effort discounting factors. Model fitting and comparison (two-learning rate *vs*. two-temporal discounting) were performed. Principal Component Analysis and linear regression identified factors predicting amotivation severity. Correlations of estimated factors with nucleus accumbens and anterior insular cortex (AIC) functional connectivity were analyzed.

**Results:**

Overall, greater 2-back than 0-back accuracy occurred in longer, positively incentivized tasks. Non- or minimally depressed individuals showed accuracy difference by N-back load at higher rewards, with divergence between reward and loss tasks at higher incentive and longer lengths. The two-temporal discounting model best explained these results. Cognitive effort discounting specifically predicted amotivation scores, derived from BDI and AES, and correlated with AIC-anterior mid cingulate cortex (aMCC) functional connectivity.

**Conclusions:**

Our findings demonstrate amotivation is specifically associated with cognitive effort devaluation in a cost-benefit analysis incorporating loss aversion, incentive learning, temporal discounting, and cognitive effort discounting. Modulation of effort valuation via the AIC-aMCC network suggests a potential treatment target.

## 1 Introduction

Major depression is characterized by a marked decrease in interest or pleasure in most or all activities. It can lead to motivational deficit, which is associated with functional impairment in patients with major depressive disorder (1). These symptoms of apathy, anhedonia, and amotivation are related to and can arise from impairment in any components of goal-directed behavior, including learning of rewards, approach-related behaviors, and willingness to exert effort to obtain rewards (2). Particularly, motivation facilitates overcoming the cost of an effortful action to achieve the desired rewarding outcome (3).

Preference to choose approach/avoidance and effortful behavior are implicitly learned through reinforcement. Reward learning, which may involve separate neural pathways for approach and avoidance behavior, occurs in both positive and negative outcomes (4). Reward incentives can also boost attentional effort and conversely, effort can discount choice values in decision-making modulated by dopamine (5–7). Individuals typically place a higher value on effort when it is directed towards avoiding punishment rather than obtaining rewards (8, 9). Thus, intrinsic motivation for loss aversion is greater particularly for effortful goal-directed behavior. Discounting of learned reward values is sensitive to time delay and reward magnitude (10, 11). Additionally, temporal discounting of learned values is less steep for losses than rewards, and the magnitude effect on discounting in rewarding outcomes is absent in monetary loss outcomes (12, 13).

Reward-based decision making involves the dopaminergic pathway-projected brain regions including the nucleus accumbens (NAc), caudate, putamen, orbitofrontal cortex, anterior insula (AIC), anterior and posterior cingulate cortex (14). Particularly, the NAc and AIC are involved in evaluating effort. The NAc is more active when making rewarding choices requiring less physical effort, while the AIC is associated with devaluing effortful options (15, 16). Moreover, variability in dopamine responses in the NAc is associated with willingness to exert effort for larger, low-probability rewards, whereas such variability in the insula is negatively correlated with unwillingness to exert effort for rewards (7).

A meta-analysis of behavioral data using a reinforcement learning framework revealed that anhedonia and major depressive disorder were associated with diminished reward sensitivity but not impaired learning (17). Considering that effort enhances both reward and loss sensitivity to outcomes (18) and can devalue choice options in decision-making (6, 7), effort expenditure may be critical factor in the reinforcement learning processes of depressed individuals. Indeed, studies of effort-based decision-making have demonstrated that individuals with major depressive disorder are less inclined to exert effort for rewards (19, 20). Moreover, another study indicated that these patients exhibit reduced effort in both reward acquisition and loss avoidance, despite intact anticipation of negative outcomes (9). Consequently, the neurocognitive underpinnings of anhedonia and amotivation in depression encompass effort cost valuation. However, the extent to which different dimensions of effort cost, such as exertion time and magnitude, are differentially impacted in depression remains unclear.

To examine the implicit neural processing of cost-benefit analysis, we developed and conducted a monetary incentive n-back task and employed resting-state fMRI. We manipulated cognitive load, incentive valence, incentive magnitude and task length to investigate the influence of integrated valuation on cognitive performance and associated brain networks. Integrating reward and effort-based valuation models, we aimed to identify the computational mechanisms and the neural network underlying cost-benefit valuation, and to identify the components associated with amotivation. We hypothesized that cognitive effort discounting is linked to amotivation and explored the relationship between model-derived parameters and functional connectivity within the NAc and AIC, key valuation processing hubs.

## 2 Materials and methods

### 2.1 Participants and procedure

Forty-nine participants consented to the procedures approved by the Institutional Review Board of International St. Mary’s Hospital. Participants aged 19 to 65 years were included in the study. Exclusion criteria encompassed individuals with a history of major psychiatric disorders, neurological disorders, acute or severe physical illnesses requiring hospitalization or surgery, alcohol or substance abuse disorders, or neurodevelopmental disorders. In addition, any individuals who were on any CNS drugs within the past 2-weeks were excluded. We screened for potential alcohol use disorder using the Alcohol Use Disorder Identification Test (AUDIT), with a score greater than 15 indicating harmful alcohol consumption (21). The self-rating scales including the Beck Depression Inventory – II (BDI) (22, 23) and the Apathy Evaluation Scale (AES) (24, 25) were used to measure the severities of depressive symptoms and amotivation in all participants. All participants performed a monetary incentive N-back task on a laptop computer and then underwent a brain scan including structural and functional Magnetic Resonance Imaging (MRI).

### 2.2 Monetary incentive N-back task

We modified a prior task (9) by incorporating a n-back task to examine the implicit effects of incentive valence, incentive magnitude, and costs of temporal delay and cognitive effort on cognitive performance.

**Figure 1:**
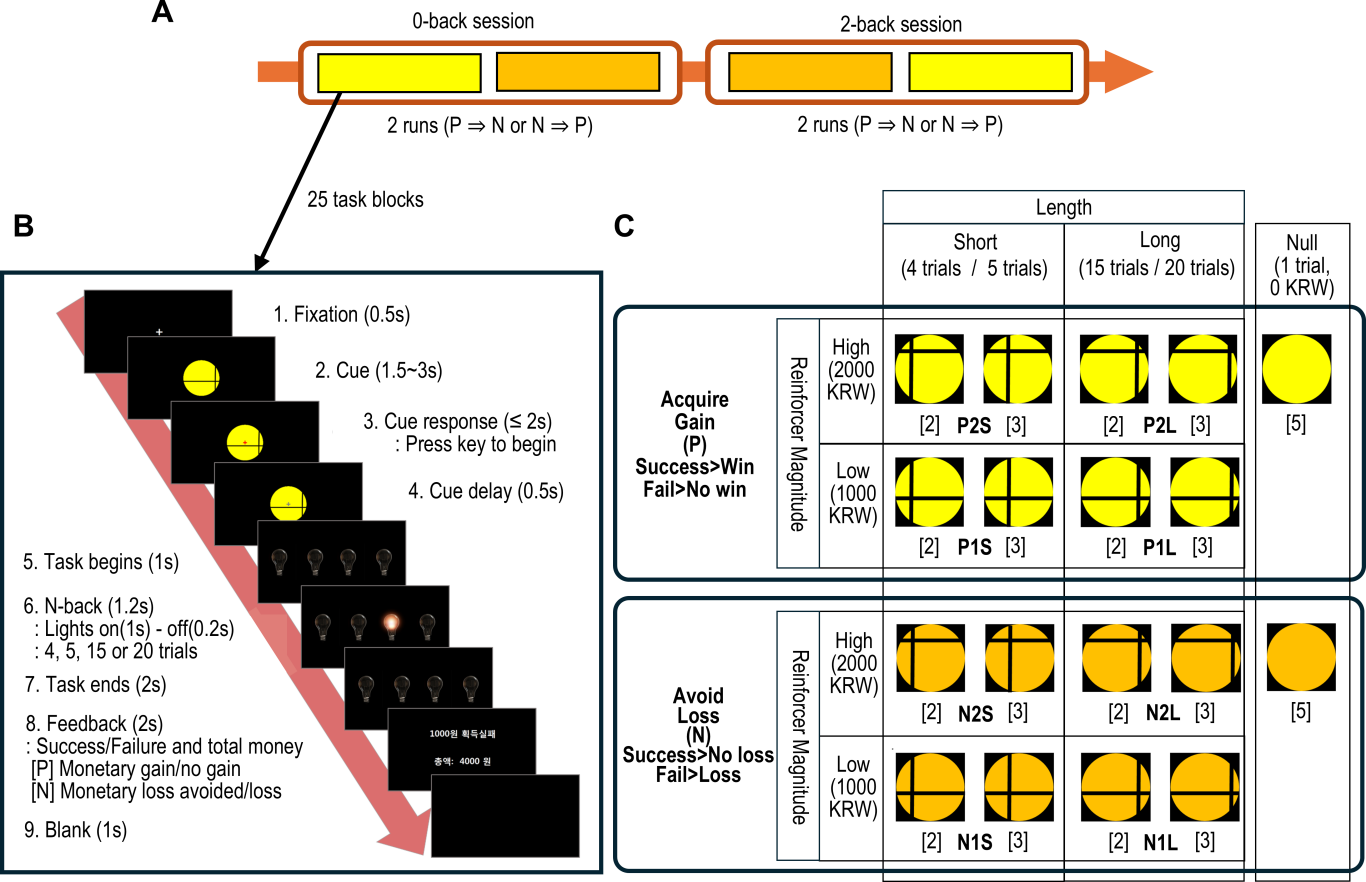
The schematic description of the monetary incentive N-back task. **(A)** The task was performed sequentially from a 0-back to a 2-back session. Each session consisted of a reward (positive incentive) and a loss (negative incentive) run in a pseudo-randomly counterbalanced order across participants. **(B)** A single task block consisted of a cue presentation, the n-back trials and performance feedback. **(C)** The cues indicated the combination of task block conditions defined by 2 incentive valences (i.e., reward and loss), 2 incentive magnitudes as monetary value in South Korean Won (KRW) (i.e., high and low), and 2 task lengths (i.e., short and long). Additionally, null conditions were denoted by 2 cues. The number of blocks per cue is specified in brackets. Each condition comprised 5 n-back task blocks.

The task was conducted in two sessions, in the order of 0-back and 2-back. Each session comprised one positive and one negative incentive run, and their order was counterbalanced across participants. In positive incentive runs, participants earned monetary rewards for successful completions and received no reward for unsuccessful ones. Conversely, in negative incentive runs, they avoided monetary losses for successful completions and incurred losses for unsuccessful ones (**Figure 1A**).

The task began with the presentation of a cue that indicated the condition for the current task block. Participants then pressed a start key to initiate the trial. Subsequently, four light bulbs were displayed, one of which illuminated randomly and consecutively. Participants were required to press a key corresponding to the currently illuminated bulb for the first session (0-back), or the bulb lit two sequences prior for the second session (2-back). Following the completion of 4 to 20 trials in each n-back block, feedback of money earned/loss avoided after successful performance, or no money earned/money lost after failed performance was presented (**Figure 1B**).

The n-back blocks varied in task length (i.e., short or long trials) and incentive magnitude (i.e., high or low). A single task run comprised a randomized sequence of 20 n-back blocks (each with a fixed incentive type and n-back task) and 5 null blocks (each consisting of a single trial without any contingencies) (**Figure 1C**).

Participants received instructions on n-back task procedures and were informed that the cue figure indicated the length of the task block and the magnitude of potential monetary earnings or losses avoided for successful completion. However, the specific task lengths and contingencies assigned to each cue were not explained. Participants were informed of the task load (i.e., 0-back or 2-back) at the beginning of each session. A practice session was administered to ensure participants comprehended and correctly executed the task.

After completing the task, all participants filled out a cue assessment questionnaire to ascertain the formation of subjective cue values. The post-task cue assessment questionnaire had three sections: subjective cue value assessment, cue preference within task incentive valences, and cue preference between task incentive valences. In the subjective cue value assessment, participants rated the value of individual cues using a scale of 0 to 3,000 Korean Won, in 500 Won increments. They were explicitly instructed to assign these values subjectively, independent of the actual task values. For cue preference within incentive valences, participants ranked each of the 12 cue triplets from 1 to 3. Each triplet set had identical incentive valence and included one null cue and all possible incentive cue pairs. Cue preference between incentive valences was assessed by participants selecting a preferred cue from comparisons of reward and loss task cue pairs (16 pairs total).

### 2.3 Image data acquisition

The MRI data were obtained using a SIEMENS 3.0T scanner (MAGNETOM Skyra, SIEMENS, Germany). High-resolution T1- weighted images were acquired using a 3D magnetization prepared rapid acquisition gradient echo (MP-RAGE) sequence with the following parameters: field of view = 256 mm, voxel size = 1.0×1.0×1.0 mm^3^, TR = 2300 ms, TE = 2.19 ms, flip angle = 9°. Then functional MRI (fMRI) data were acquired using T2*-weighted single-shot echo-planar imaging (EPI) sequence with the following parameters: field of view = 195 mm, voxel size = 2.5 × 2.5 × 4.0 mm^3^, number of slices = 34, TR = 2000 ms, TE = 30 ms, flip angle = 90°. Participants were instructed to keep their eyes fixed at a projected crosshair and not think about anything, while being scanned for 6 min 46 s.

### 2.4 Behavioral task data analysis

To differentiate the within-task block effects of incentive magnitude and task length, mean accuracy rates were calculated in two distinct ways. First, eight mean accuracy rates were computed for each combination of N-back load, incentive valence, and incentive magnitude, disregarding task length. Second, mean accuracy rates for each combination of N-back load, incentive valence, and task length were calculated, disregarding incentive magnitude.

Since the distribution of the n-back task accuracy rates were skewed due to ceiling effects, we conducted non-parametric tests. For within-group comparisons of task performance between conditions, Wilcoxon signed rank tests were applied separately for the mean accuracy rates by incentive magnitude and task length. The relationship of the accuracy rates with the BDI and AES scores were examined by performing Spearman’s rank correlation tests. A *post-hoc* analyses after excluding participants with BDI score greater than 13 (i.e. mild to severe depression) was conducted to clarify the conditional effects on performance unconfounded by depression. Analyses results were considered statistically significant at Bonferroni-corrected *P* < 0.05 (i.e. *P* × 8 for Wilcoxon tests; *P* × 16 for Spearman tests). Statistical tests of accuracy were performed using R Statistical Software (v4.3.1; R Foundation for Statistical Computing 2023).

### 2.5 The post-task cue assessment data analyses

The cue preference rank score was defined as the average of each cue’s rank-reversed scores derived from the within-incentive-valence cue preference data. For the between-incentive-valence cue preference data, relative cue preference was defined as the ratio of preferring reward cues over loss cues. We calculated six distinct relative cue preference ratios, each corresponding to specific combinations of incentive magnitude and task length. These included the relative preference for completely matched and mismatched reward over loss (N→P [C], N→P [IC]), low reward over high loss and high reward over low loss regardless of task length (N2→P1, N1→P2), and short reward over long loss tasks and long reward over short loss tasks regardless of incentive size (NL→PS, NS→PL). Scores ranged from 1 to 3, with higher scores indicating a greater preference for the cue.

Wilcoxon one-sample tests were performed on the subjective cue values to compare their median to the actual cue values. For the cue preference rank scores, Wilcoxon signed-rank tests were conducted to compare reward or loss cue scores of either matched incentive magnitude or task length. The significance of the relative cue preference ratio was assessed using Wilcoxon one-sample tests. Additionally, Wilcoxon signed-rank tests were used to investigate the effects of incentive magnitude and/or task length by comparing matched to other unmatched relative cue preference ratios. Wilcoxon signed-rank tests were considered statistically significant at Bonferroni-corrected *P* < 0.05 (i.e. *P* × 2 for cue preference rank scores; *P* × 5 for relative cue preference).

### 2.6 Computational model

We assumed that the intra-individual variability in the n-back task performance is modulated by the task’s anticipated value. This value is derived from a cost-benefit analysis that incorporates incentive valence and magnitude, task duration, and cognitive load. To capture these conditional components within a unified value function, we integrated three distinct models: the loss aversion model for accounting the different impact of monetary gains versus avoided losses; the temporal difference learning model for learning the reward magnitude and temporal delay associated with each cue; and the effort discounting model, which accounted for cognitive burden. Lastly, the softmax function was used to model performance as determined by cognitive capacity.

Based on the prospect theory, outcomes of “gains” and “losses” have different value functions (26, 27). When *x* is the amount of reward, the value of the reward is determined by *rho* (ρ), the degree of risk aversion, and *lambda* (*Λ*), the loss aversion factor, as follows.


λ>0

$$\text{if }x\geq 0,\;\;V_{a}\left(x\right)=x^{\rho }$$

$$\text{if }x<0,\;\;V_{a}\left(x\right)=\;-\lambda \cdot {\left(-x\right)}^{\rho }$$

We used the hyperbolic function for modeling cognitive effort (28). At time t, the value is obtained by the reward amount (*x*), discounting factor for cognitive effort (γ) and the cognitive load (c). Values for c was 0 for 0-back session and 2 for 2-back session. The γ values ranged between 0 and 1.


$$V_{c}\left(t\right)=x\cdot \frac{1}{\left(1+\gamma \cdot \text{c}\right)}$$

Then we assumed *ρ* = 1 for simplicity and combined the reward and effort value functions by substituting the *x* in the cognitive effort model with the reward value function, *V_a_(x)*. Thus, the trial cue stimulus value at n-back turn *t* (i.e., *V(S_t_)*) is formulated as follows.

For positive incentive (*i*.*e*., *x* ≤ 0),


$$V(S_{t})=\;\frac{V_{a}(x)}{\left(1+\gamma \times \text{c}\right)}\;=\;\frac{V\left(S_{t-1}\right)}{\left(1+\gamma \times \text{c}\right)}$$

For negative incentive (*i*.*e*., *x* > 0),


$$V(S_{t})=\;\frac{V_{a}(x)}{(1+\gamma \times \text{c})}\;=\;\frac{\lambda \cdot \;V\left(S_{t-1}\right)}{\left(1+\gamma \times \text{c}\right)}$$

The cue stimulus value is further updated by reward received, learning rate (*α*), and temporal discounting factor (*κ*) by the temporal difference learning model (29). The *α* and *κ* values ranged between 0 and 1.


$$\;V^{\prime }\left(S_{t}\right)\leftarrow V(S_{t-1})+\alpha \times \left(r+\left(\kappa \times V(S_{t})\right)-V(S_{t-1}\right))$$

Given prior reports of the differential impairments in reward and punishment reinforcement and the reported abnormalities in delay discounting in depression, we constructed two distinct learning models (30, 31): one incorporating dual learning rate factors (*α1* and *α2*) and another featuring dual temporal discounting factors (*κ1* and *κ2*).

We assumed that the probability of correct response was determined by the cognitive capacity (*β*) and the cue stimulus value before receiving reward. The softmax function was modified to incorporate 4 response choices so that random response would result in a probability of 25% (i.e., when *β* = 0) as follows.


β>0

$$P(S_{t})=\;\frac{1}{\left(1+3\times e^{\left(-\beta \cdot V(S_{t})\right)}\right)}$$

### 2.7 Model fitting and comparison

Two models were compared; the dual learning rate model (2-LR) and the dual temporal discounting model (2-TD) in which separate learning rates and temporal discounting rates were assigned for positive and negative incentive trials. (**Supplementary Methods S1** code for 2-TD & **Supplementary Methods S2**. code for 2-LR model).

We estimated the model parameters, identified individually responsible models, and performed a group-level model comparison using the hierarchical Bayesian inference (HBI; https://payampiray.github.io/cbm) (32). First, each model was fit to individual subject data using Laplace approximation. Then HBI was conducted for parameter estimation and to generate model frequency and protected exceedance probability. The estimate of how much each model is expressed across the group (i.e., model frequency) and the probability that each model is the most likely across the group while considering differences between model frequency arising by chance (i.e., protected exceedance probability, *P_px_*) were computed. The model with higher *P_px_* was selected for model validation.

### 2.8 Model validation

To validate the task model for clinical amotivation, we performed a multiple linear regression to examine whether the parameters from the winning model (higher *P_px_*) can predict the dependent variable representing clinical amotivation. First, a Principal Component Analysis (PCA) was conducted to extract the general component scores representing the overlapping clinical construct of amotivation from the BDI and AES scores. Following standardization (centering and scaling) of the BDI and AES scores, PCA was performed on the correlation matrix. The amotivation component was identified if a principal component met Kaiser’s criterion (Eigenvalue > 1) and contributed to a cumulative proportion of variance of at least 80%. For interpretability, only BDI and AES scores with positive loadings on this component were considered.

Then, we used the amotivation component scores (ACS) as the dependent variable and the five parameters (excluding *beta*, the cognitive capacity factor) from the winning model in an Ordinary Least Squares (OLS) Linear Regression. The model equation for the regression included ACS for the i-th individual(*y_i_*), intercept (*β_0_*), regression coefficients for the winning model parameters (*β_1~5i_*), values for the winning model parameters(*x_1~5i_*), and the error term or residual (∈_i_) is as follows.


$$y_{i}=\;\beta_{0}+\;\beta_{1}\cdot x_{1i}+\;\beta_{2}\cdot x_{2i}+\;\beta_{3}\cdot x_{3i}+\;\beta_{4}\cdot x_{4i}+\;\beta_{5}\cdot x_{5i}+{Є}_{i}$$

All model validation procedures were conducted using R Statistical Software (v4.3.1; R Foundation for Statistical Computing, 2023). PCA was performed using the *prcomp* function, and OLS linear regression was carried out using the *lm* function. To address potential heteroscedasticity, robust standard errors were calculated using the HC3 estimator, implemented via the *coeftest* function (from the *lmtest* package) in conjunction with the *vcovHC* function (from the *sandwich* package).

### 2.9 Image data analysis

Preprocessing and functional connectivity analysis of the fMRI data were conducted using the CONN toolbox (conn v.21.a, http://www.nitrc.org/projects/conn, RRID: SCR: 006394) and SPM12 (RRID: SCR:007037) procedures. Realignment and unwarp procedures were conducted in which all scans were coregistered and resampled to the first scan as a reference image to adjust head motion and match the deformation field. Next, slice-timing differences were corrected, and outlier scans were identified. Functional and anatomical data were normalized into standard MNI space and segmented into grey matter, white matter, and CSF tissue classes. Lastly, functional data was spatially smoothed by convolution with a 6 mm full-width-at-half-maximum Gaussian kernel.

In the denoising step, an anatomical component-based noise correction method (aCompCor) was implemented to regress out noise components from the white matter and cerebrospinal fluid areas, estimated subject-motion parameters, identified outlier scans and quality assurance metrics. Then despiking was conducted to remove artificially high signals and temporal band-pass filter at 0.001~0.1Hz was applied to specifically focus on slow-frequency fluctuations. Subsequently, seed-to-whole brain (seed-to-voxel) correlation maps were computed using seeds in the anterior insular cortex (AIC) selected from the salience network defined by CONN’s independent component analyses of the Human Connectome Project dataset. Additionally, the nucleus accumbens (NAc), defined by the Harvard-Oxford atlas and generated using FSLeyes (https://git.fmrib.ox.ac.uk/fsl/fsleyes/fsleyes), was also used as a seed in the first-level analyses.

In the 2^nd^-level analyses, regression analyses with each model parameter as covariate across all subjects were conducted examining the seed-to-voxel functional connectivity arising from the four seeds in the bilateral AIC and the NAc. First, F-tests were conducted to find any effects among the four seeds of interest. Then, *post-hoc* T-tests were performed to specify the seed that was the main contributor to the F-test results. Results were considered statistically significant with cluster threshold at False Discovery Rate-corrected *P* (FDR-*P*) < 0.05 and voxel threshold at uncorrected-*P* < 0.001 using random field theory parametric statistics.

## 3 Results

Thirty-one females and twelve males with a mean age of 37.0 years (range 21~65, SD = 15.4) completed the study. The participants’ depression severity by BDI scores ranged from minimum to severe (0~33) with a mean score of 10.6 (SD = 9.3) and 12 participants (27.9%) were mildly to severely depressed (BDI > 13). The mean AES score was 34.9 (SD=6.26) and only two participants (4.7%) showed AES score greater than the Korean cutoff values of 45.2 (**Table 1**).

**Table 1:** Sociodemographic and clinical characteristics in all participants (n=43).

Characteristics	Number (%) or Mean
Gender : Female/Male	31/12 (72.1%/27.9%)
Age	37.0 ± 15.4 [21~65]
Years of Education	14.9 ± 2.1 [9~18.5]
Smoking : Non-smoker/Stop smoking/Smoking	38/3/2 (88.4%/7%/4.7%)
AUDIT	4.37 ± 3.57 [0~14]
Low risk (0~7) Medium risk (8~15) High risk (16~19) Addiction likely (20~40)	34 (79.1%) 9 (20.9%) 0 0
BDI	10.6 ± 9.3 [0~33]
Minimum (0~13) Mild (14~19) Moderate (20~28) Severe (29~63)	31 (72.1%) 5 (11.6%) 3 (7.0%) 4 (9.3%)
AES	34.9 ± 6.3 [24~49]
Korean Cutoff (>45.21)	2 (4.7%)

mean ± standard deviation, (%), [range] are presented.

AUDIT, Alcohol Use Disorder Identification Test; BDI, Beck Depressive Inventory; AES, Apathy Evaluation Scale.

### 3.1 Task accuracy

**Figure 2:**
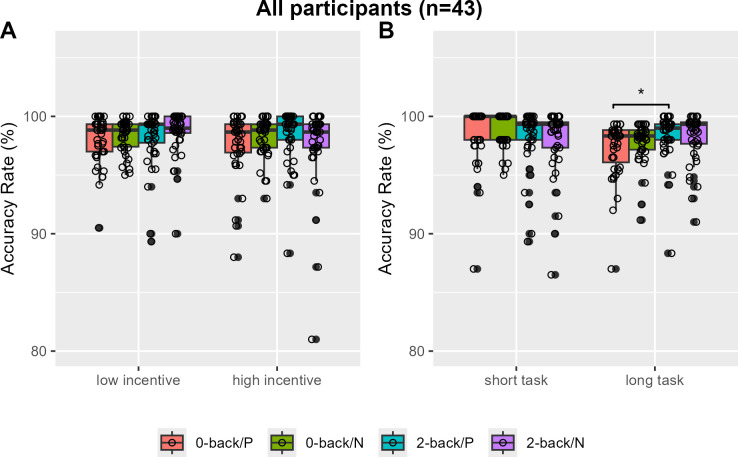
Accuracy rates of the monetary incentive n-back task in all participants. Accuracy rates are compared by N-back and incentive valence in low and high magnitude conditions **(A)** and in short and long task length conditions **(B)**. P, positive incentive (reward); N, negative incentive (loss); Bonferroni-corrected *P <0.05.

**Figure 3:**
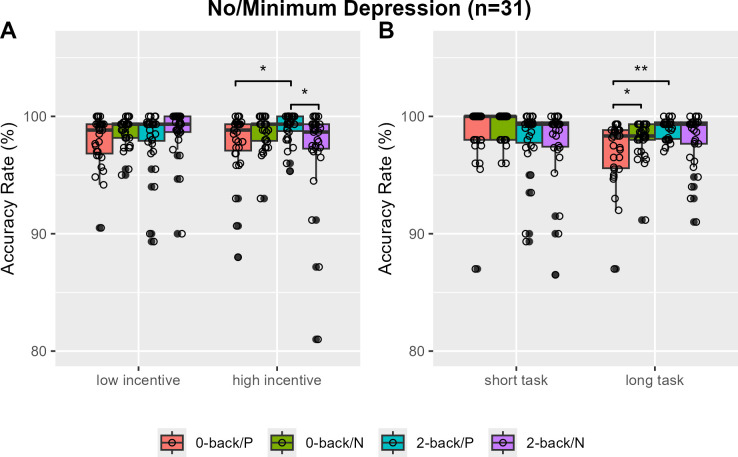
Accuracy rates of the monetary incentive N-back task in individuals with no or minimal depression. Accuracy rates are compared by N-back and incentive valence in low and high magnitude conditions **(A)** and in short and long task block length conditions **(B)**. P, positive incentive (reward); N, negative incentive (loss); Bonferroni-corrected *P <0.05, **P<0.001.

The 2-back task showed a significantly higher overall accuracy rate compared to the 0-back task, with median rates of 0.983 (IQR = 0.020) and 0.985 (IQR = 0.022), respectively (V=175, *P*=0.0016). Specifically, no accuracy rate differences by N-back load or incentive valence were found in low and high incentive trials (**Figure 2A**). However, greater 2-back than 0-back task accuracy rates were observed only with positively reinforced longer trials (V=202, Bonferroni-corrected P=0.0250, Cohen’s d=-0.357) (**Figure 2B**). When analysis was restricted to individuals with no or minimum depression (n=31), the accuracy rates of the 2-back task were greater than that of the 0-back task in positive incentive trials with higher reward or longer trials (V=56, Bonferroni-corrected *P*=0.0115, Cohen’s *d*=-0.572; V=43, Bonferroni-corrected *P*=0.0008, Cohen’s *d*=-0.761) (**Figures 3A, B**). Furthermore, accuracy was higher in 2-back trials featuring positive incentives of higher magnitude, in contrast to trials involving negative incentives (V=349.5, Bonferroni-corrected *P*=0.0353, Cohen’s d=0.481). Conversely, accuracy was lower in long 0-back trials under conditions of positive incentives compared to negative incentives (V=72.5, Bonferroni-corrected *P*=0.0424, Cohen’s *d*=-0.522) (**Figures 3A, B**).

**Figure 4:**
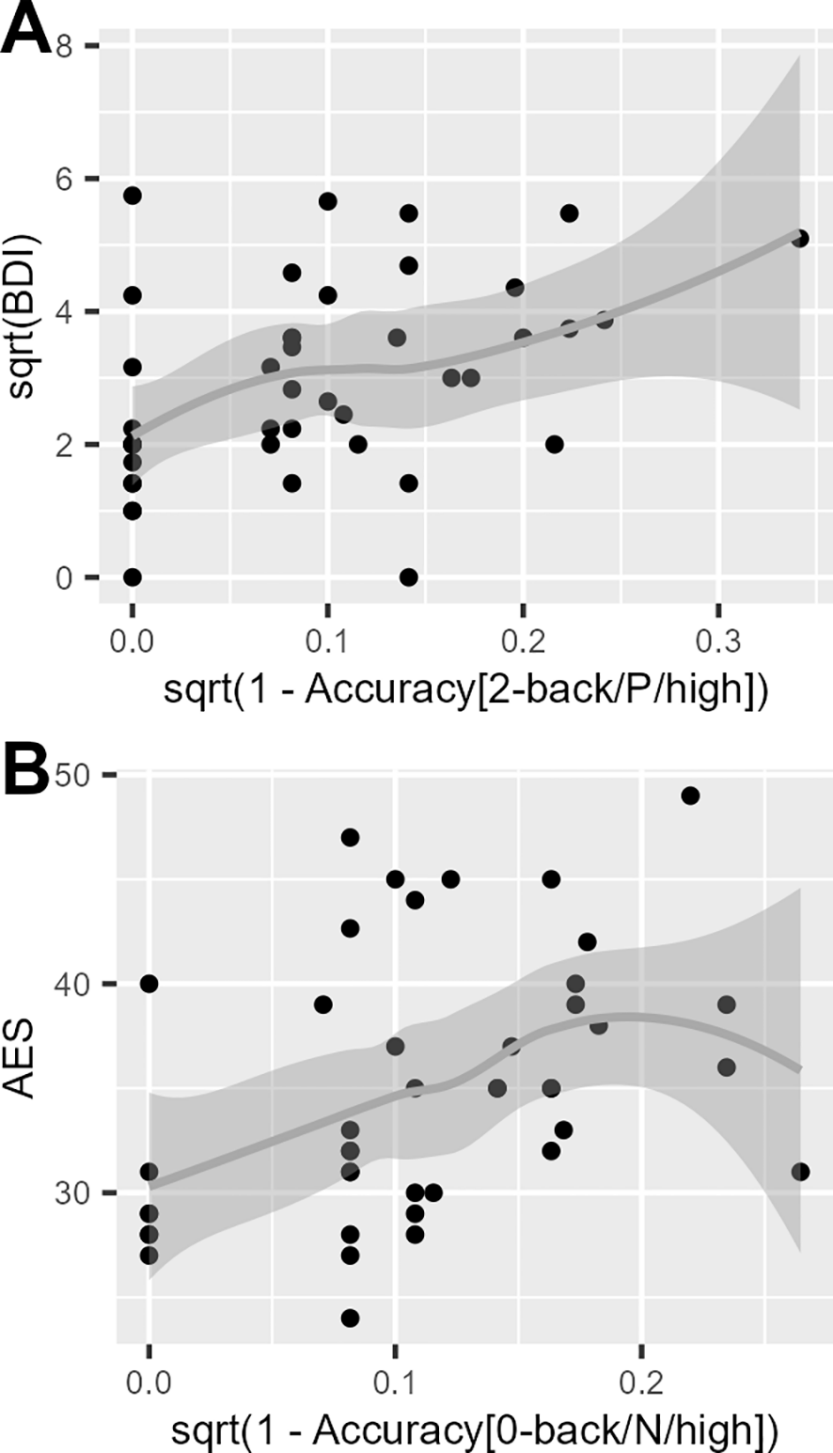
Scatter plots with the trend fitted lines (i.e. grey solid lines) using locally estimated scatterplot smoothing showing the association of the accuracy of the 2-back task with the Beck Depression Inventory (BDI) and Apathy Evaluation Scale (AES) scores. The transformed (i.e. square root, sqrt) accuracy values of the 2-back task blocks with high reward (positive incentive) magnitudes **(A)**, and high loss (negative incentive) magnitude **(B)** are presented for better visualization. **(A)** Spearman’s *rho* = -0.460, Bonferroni-corrected *P* = 0.031; **(B)** Spearman’s *rho* = -0.457, Bonferroni-corrected *P* = 0.033.

BDI scores were significantly associated with accuracy rates in the 2-back task involving positive incentives of high magnitude, showing a moderate effect size (**Figure 4A**). Conversely, AES scores correlated with accuracy rates in the 0-back task with negative incentives of high magnitude, showing a moderate effect size (**Figure 4B**). AUDIT scores did not correlate with any of the task accuracy rates.

### 3.2 Post-task cue valuation and preference

**Figure 5:**
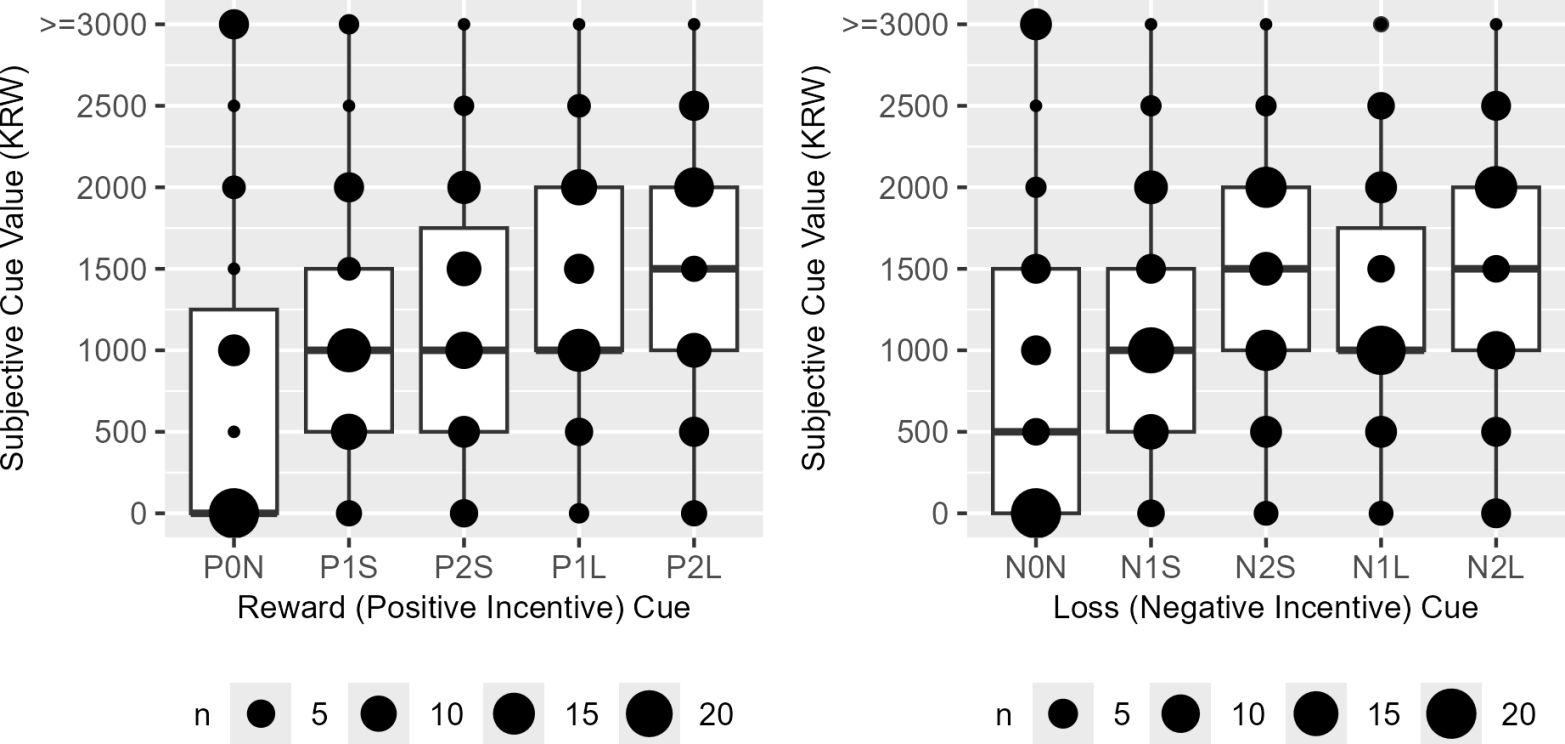
The boxplots of the reward (P) and loss (N) cue subjective values in Korean Won (KRW). P0N/N0N, null cues; P1S/N1S/P1L/N1L, 1000 KRW incentive cues; P2S/N2S/P2L/N2L, 2000 KRW incentive cues; P1S/N1S/P2S/N2S, short task cues; P1L/N1L/P2L/N2L, long task cues.

The subjective cue values exhibited a distribution symmetric around their actual incentive magnitude only when reward and loss cues were associated with short trials and low incentive magnitudes (P1S, V = 207, *P* = 0.421; N1S, V = 246, *P* = 0.165); in all other conditions, a significant difference in distribution was observed (P0N, V = 190, *P* < 0.001; P2S, V = 30, *P* < 0.001; P1L, V = 313, *P* = 0.003; P2L, V = 49, *P* < 0.001; N0N, V = 276, *P* < 0.001; N2S, V = 26, *P* < 0.001; N1L, V = 222, *P* = 0.038; N2L, V = 44, *P* < 0.001) (**Figure 5**).

**Figure 6:**
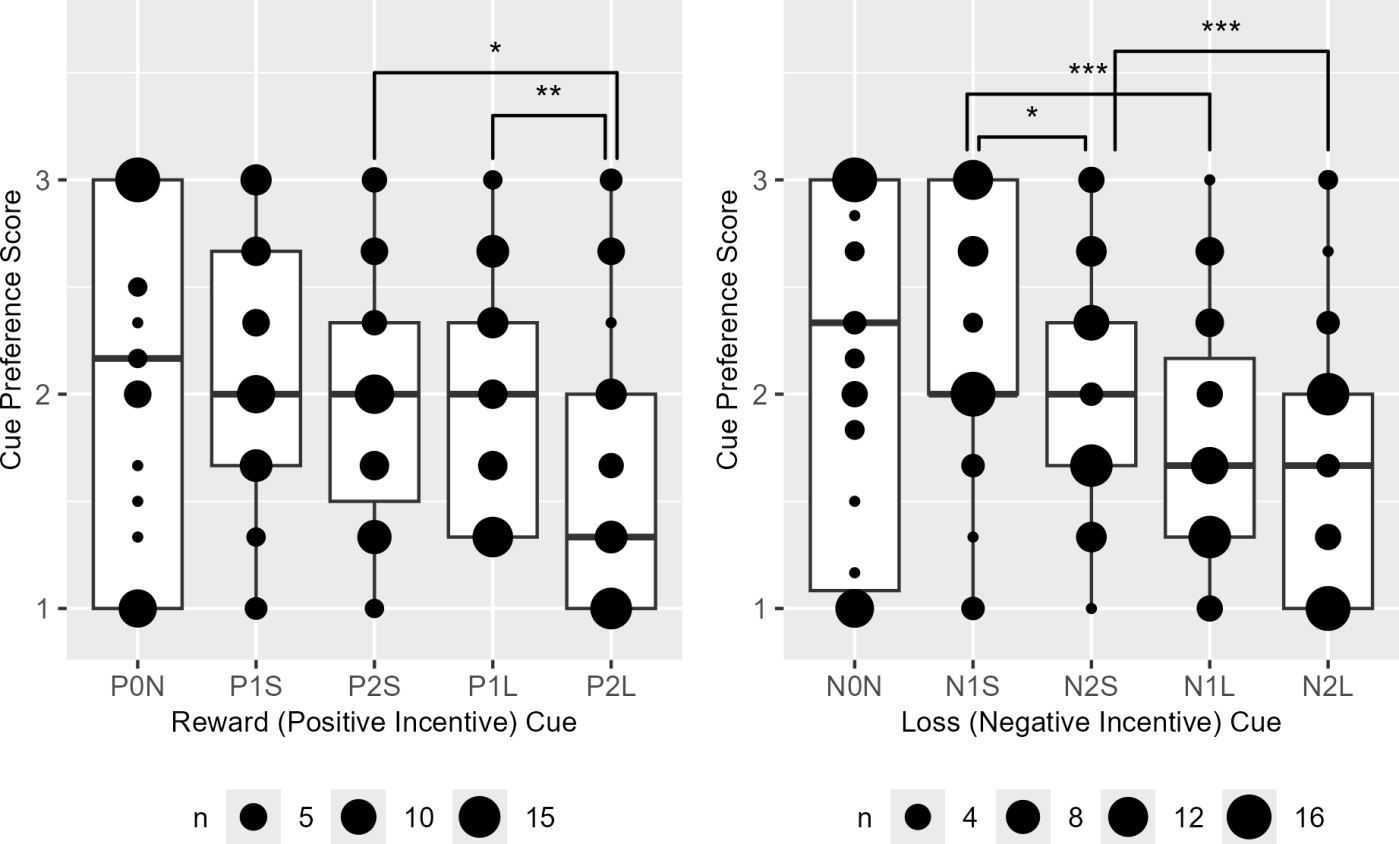
The boxplots of the reward (P) and loss (N) cue preference rank scores. P0N/N0N, null cues; P1S/N1S/P1L/N1L, 1000 KRW incentive cues; P2S/N2S/P2L/N2L, 2000 KRW incentive cues; P1S/N1S/P2S/N2S, short task cues; P1L/N1L/P2L/N2L, long task cues. Significant difference was observed in P1L > P2L (V = 604), P2S > P2L (V=511), N1S > N2S, (V=631), N1S > N1L (V=782) and N2S > N2L (V=708). Bonferroni-corrected *P* < *0.05, **0.01, ***0.001.

Among the reward cues, preference scores were significantly higher for cues associated with shorter trials delivering high rewards (P2S > P2L) and long trials offering lower rewards (P1L > P2L). Conversely, for loss incentive cues, higher preference scores were observed for cues representing short trials with lower losses (N1S > N2S), shorter trials incurring low losses (N1S > N1L), and shorter trials with high losses (N2S > N2L) (**Figure 6**).

**Figure 7:**
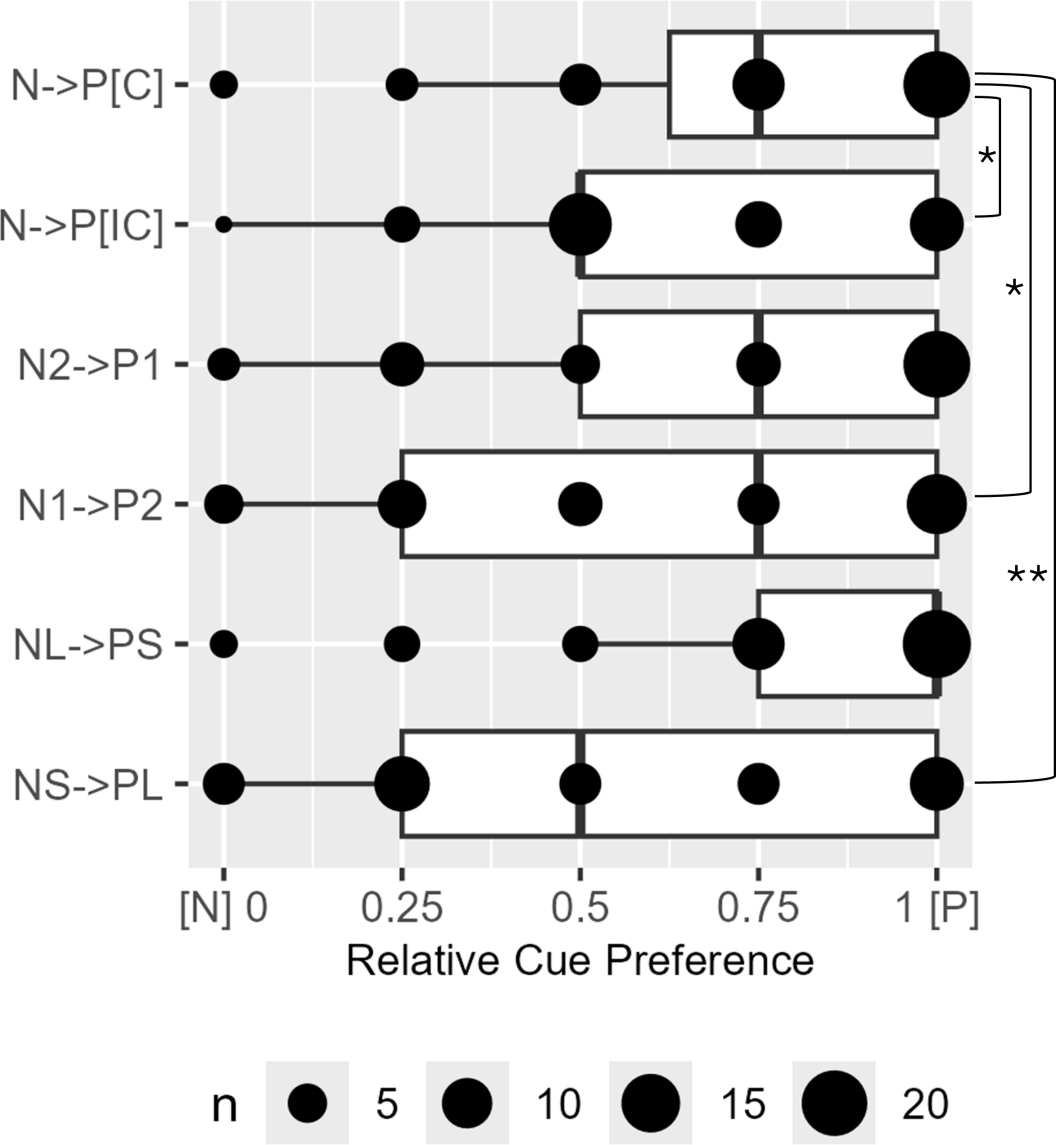
The boxplots of relative cue preference for reward (P) than loss (N) cues. [C]/[IC], compatible/incompatible cues with matched/mismatched incentive magnitudes and task lengths; P1/N1, 1000 Korean Won; P2/N2, 2000 Korean Won; PL/NL, long trials; PS/NS, short trials. Significant differences from N→P[C] were observed in N→P[IC] (V = 189), N1→P2 (V = 219), and NS→PL (V = 288). Bonferroni-corrected *P* < *0.05, **0.001.

Overall cues associated with reward incentives were significantly preferred over those with loss incentives regardless of incentive magnitudes and task lengths with the exception when the task was shorter in loss cues than in reward cues (N→P [C], V = 629, *P* < 0.001; N→P [IC], V = 280, *P* = 0.001; N2→P1, V = 609, *P* < 0.001; N1→P2, V = 464, *P* = 0.034; NL→PS, V = 693, *P* < 0.001; NS→PL, V = 402, *P* = 0.437). The distribution of reward-over-loss cue preference became significantly less skewed toward reward when incentive magnitudes and task lengths were mismatched, when incentive magnitude was lower in loss than reward cues and when task lengths were shorter in loss than reward cues (**Figure 7**).

### 3.3 Model comparison

The two-temporal discounting (2-TD) model was the most likely model across the group as compared to the two-learning rate (2-LR) model (*P_px_* = 1 for 2-TD model and *P_px_* = 3.387 × 10–^12^ for 2-LR model; model frequency 97.67% in 2-TD and 2.33% in 2-LR). The estimated responsibility and the log evidence of each model generated in each individual dataset are shown in **Supplementary Table 1**. The median value of the parameter estimates from the two-temporal discounting model are presented in **Supplementary Table 2**.

### 3.4 Model validation: OLS linear regression for amotivation component score

The PCA revealed two principal components (PC). PC1 accounted for 81.0% of the total variance with an Eigenvalue greater than 1, while PC2 accounted for 19.0% of the total variance. Together, these two components explained 100% of the total variance in the BDI and AES scores. PC1 showed strong positive loading from both BDI (0.707) and AES (0.707) suggesting that this component represents a general construct related to both depression and apathy severity. PC2, on the other hand, showed a positive loading for BDI and negative loading for AES, indicating a discordant component. Therefore, PC1 was identified as the amotivation component score (ACS) for the regression analysis (**Table 2**).

**Table 2:** Result of the principal component (PC) analysis for beck depression Inventory (BDI) and apathy evaluation scale (AES) scores.

Statistics	PC1	PC2
Eigenvalue	1.620*	0.380
Proportion of variance	0.810	0.190
Cumulative proportion	0.810	1.000
BDI loading	0.707	0.707
AES loading	0.707	-0.707

*Kaiser’s criterion: Eigenvalue > 1.

The full regression model was statistically significant (F(5,37) = 3.108, *P* < 0.05), indicating that the *alpha, lambda, gamma, kappa1, kappa2* parameters collectively explain a significant portion of the variance in the ACS. Moreover, 20% of the variance in the ACS could be accounted for by the five independent variables in the model (Adjusted R^2^ = 0.201). Among the five independent variables, only *gamma* (cognitive effort discounting factor) emerged as a statistically significant predictor of the ACS by an increasing it by 0.45 standard deviations, holding other variables constant (β* = 0.451, robust SE = 0.806, T = 2.685, *P* < 0.05) (**Table 3**).

**Table 3:** The ordinary least squares linear regression results for the principal component score of the beck depression scale and apathy evaluation scale.

Independent variable	Regression coefficient $\hat{\beta }$	Robust standard error	Standardized regression coefficient β*	T (*P*)
Intercept	-1.294	1.709	-5.188 × 10^-17^	-0.757 (0.454)
*Alpha* (learning rate)	-1.107	3.052	-0.058	-0.363 (0.719)
*Lambda* (loss aversion)	-0.830	1.262	-0.142	-0.658 (0.515)
*Gamma* (cognitive effort discount)	2.165	0.806	0.451	2.687 (0.011)*
*kappa1* (PR temporal discount)	1.583	1.047	0.227	1.513 (0.139)
*kappa2* (NR temporal discount	-0.306	1.396	-0.045	-0.219 (0.828)

PR, positive reinforcement; NR, negative reinforcement.

Model fit statistics (N=43): Adjusted R^2^ = 0.201, F (5,37) = 3.108, *P* = 0.02.

*Significance level at *P* < 0.05.

### 3.5 Functional connectivity

Multivariate analyses (F-tests) revealed that the seed-to-voxel functional connectivity originating from the AIC and NAc covaried with *alpha*, *lambda*, and *gamma* parameters. Specifically, the learning factor (*alpha*) covaried functional connectivity to the posterior lobe of the cerebellum and the cuneal cortex extending to the occipital pole. The loss aversion factor (*lambda*) showed covariance with functional connectivity to the right posterior superior temporal gyrus, the juxtapositional lobule cortex (or supplementary motor area), the central opercular cortex, and the precentral gyrus. Furthermore, the cognitive effort discounting factor (*gamma*) covaried with functional connectivity to the anterior mid-cingulate cortex. In contrast, the cognitive capacity (*beta*) and temporal discounting factors (*kappa1* and *kappa2*) did not exhibit any significant effects (**Table 4**).

**Table 4:** The seed-to-voxel functional connectivity analysis results of the model parameter effects.

Model parameter	Clusters	Coordinate (x, y, z)	F (4, 38)	Size	Size *P*-FDR	Peak *P*-uncorrected
*Alpha*	Cerebellum, posterior lobe (crus2)	38, -68, -50	6.75	80	0.00986	<0.00001
	Cuneal Cortex-Occipital Pole	-10, -88, 22	7.87	316	<0.00001	<0.00001
*Beta*	NSC					
*Lambda*	Posterior Superior Temporal Gyrus	50, -14, 8	11.72	161	0.00022	0.00002
	Juxtapositional Lobule Cortex (SMA)	6, -4, 54	9.81	141	0.00033	0.00005
	Central Opercular Cortex	52, 2, 6	9.27	113	0.00106	0.00002
	Precentral Gyrus	2, -18, 52	7.05	54	0.03910	0.00004
*Kappa1*	NSC					
*Kappa2*	NSC					
*Gamma*	Anterior Mid-Cingulate Cortex	4, 16, 30	5.52	103	0.006652	0.00002

Clusters showing significant model parameter effect on functional connectivity originating from any of the four seeds in the bilateral anterior insular cortex and nucleus accumbens are shown.

Cluster size threshold of *P*-False Discovery Rate (FDR) corrected < 0.05 and peak threshold of uncorrected-*P* < 0.001 are considered statistically significant.

NSC, no significant clusters; SMA, supplementary motor area.

**Figure 8:**
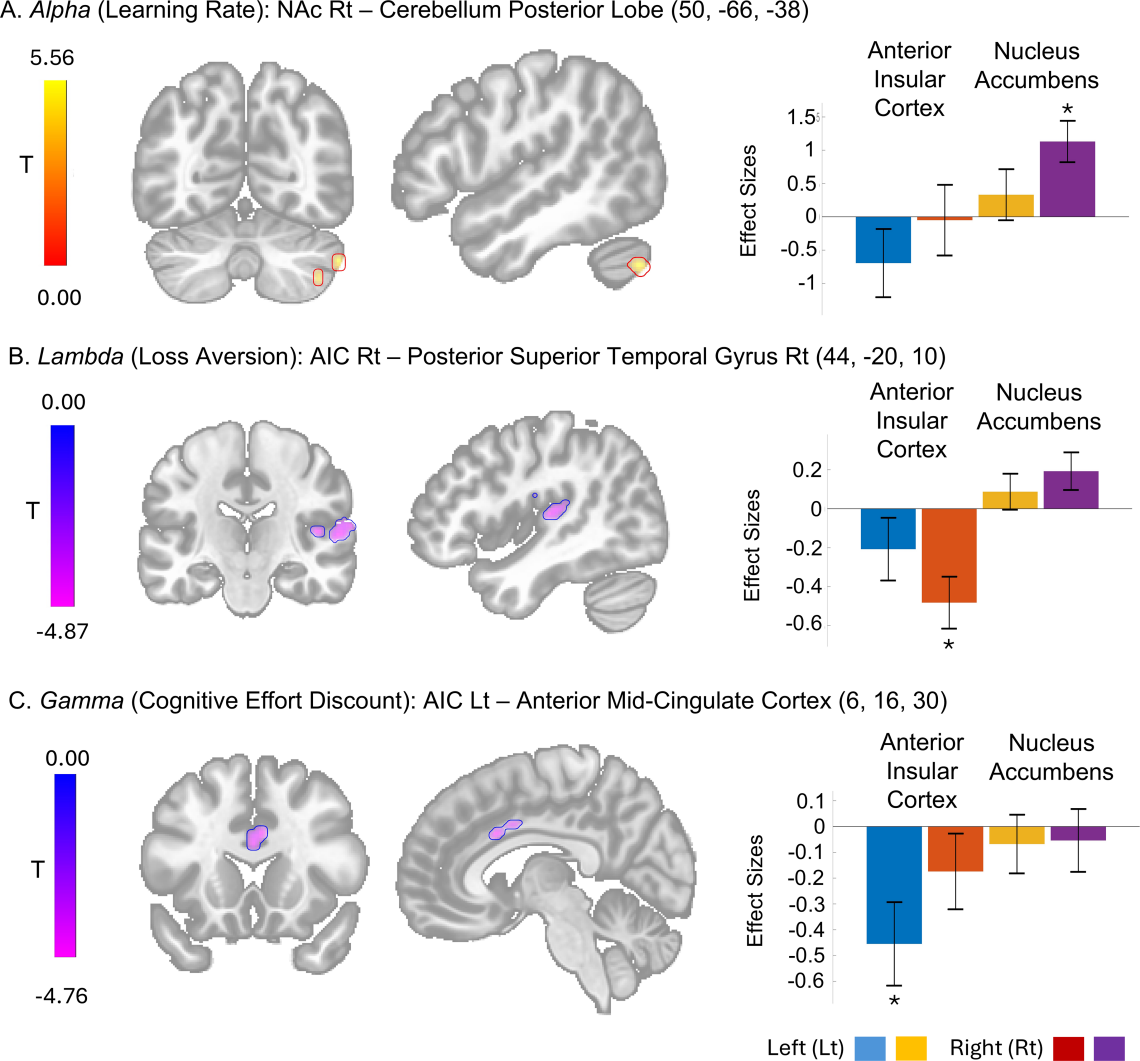
The seed-to-voxel functional connectivities with model parameter effects and their effect size. Cluster with significant effect from the T-tests of specific seed of interest including the bilateral anterior cingulate cortex (AIC) and nucleus accubmens (NAc) at cluster level False Discovery Rate-corrected *P* < 0.05 and voxel level uncorrected *P* < 0.001 are shown. The effect sizes as Fisher transformed correlation coefficient and their error bars representing 90% confidence intervals are presented. *The bar graphs representing the statistically significant effect size [**(A)** size = 131, T(41) = 6.12; **(B)** size = 272, T(41) = -6.08; **(C)** size = 97, T(41) = -4.73].

The *post-hoc* t-tests revealed that the learning rate factor (*alpha*) positively correlated with functional connectivity between the right NAc and the posterior lobe of the cerebellum (**Figure 8A**). In contrast, the loss aversion factor (*lambda*) negatively covaried with functional connectivity between the right AIC and the right posterior superior temporal gyrus (**Figure 8B**). The cognitive effort discounting factor (*gamma*) also showed a negative correlation with functional connectivity between the left AIC and the anterior mid-cingulate cortex (aMCC) (**Figure 8C**).

The AUDIT score did not show significant effect on the functional connectivity originating from the seeds of interest.

## 4 Discussion

We initially established the validity of the monetary incentive n-back task in differentiating the interactive effects of incentive valence and magnitude, task length, and cognitive load. Task trials with greater cognitive load exhibited enhanced performance when reinforced with higher positive incentives or during longer tasks. The divergent performance outcomes observed with positive and negative incentives were mediated by the incentive magnitude and the task length. The conditional effects became evident only after individuals with depression were excluded from the behavioral data. Additionally, the formation of subjective cue values through task performance was evident in the varied cue preference across both reward and loss tasks and the reduced reward-over-loss cue preference linked to incentive magnitude and task length. Subsequently, we demonstrated that a computational model incorporating temporal difference learning, loss aversion, cognitive effort discounting and dual temporal discounting factors accounted for the variability in the n-back task performance across conditions of incentive valence, incentive magnitude, task length, and cognitive load. Finally, among the model parameters, the cognitive effort discounting factor accounted for the variance in clinical amotivation severity as hypothesized. Moreover, in partial support of the model’s neural underpinnings, learning rate, loss aversion, and cognitive effort discounting were associated with distinct neural networks originating in the NAc and AIC.

The effort-based decision-making paradigm has been employed to neurobehaviorally operationalize motivation. Previous research examining the influence of reward and effort values on preferences has demonstrated an association between depression and reduced willingness to exert effort for reward, consistent with anticipatory anhedonia (20, 33, 34). Moreover, a recent study suggests that expenditure of effort and reward sensitivity may be independently linked to anhedonia (35). Computational modeling studies have revealed that elevated sensitivity to effort cost is associated with depression across preference, performance, and learning tasks, with individuals with major depressive disorder exhibiting a steeper discounting of rewards in response to cognitive effort (36, 37). While these studies explicitly assessed willingness to exert effort through choice-based paradigms, our investigation examined the implicit impact of reward and effort on cognitive performance, yielding congruent yet extended findings. Specifically, the cognitive effort discounting factor, within the valuation process affecting cognitive performance variability, emerged as the key correlate of amotivation, as opposed to the loss aversion, learning or temporal discounting factors. These results indicate that the detrimental effects of heightened effort devaluation extend beyond decision-making to influence behavioral performance, potentially contributing to the concentration difficulties observed in depression.

The observed functional connectivity patterns associated with loss aversion and learning rate align with previous research findings. The loss aversion-related functional connectivity centered on the AIC corroborates prior studies implicating the AIC in aversive conditioning and decisions to avoid imminent loss (38, 39). Given the established role of the right posterior superior temporal gyrus (pSTG) in serial visual feature search and object identification involving complex goal-directed movement (40, 41), the AIns-right pSTG connectivity likely reflects the process of assigning or identifying aversive value from visual cue features.

Learning rate, a critical parameter in reinforcement learning models, has been extensively linked to prediction error. Previous animal and human studies have demonstrated that learning rate modulates reward prediction error signals originating from midbrain dopaminergic projections to the NAc (42–44). Notably, our findings revealed learning rate-associated NAc connectivity to the cerebellum. Prior clinical and animal research has established the cerebellum’s role in reward-based reversal learning and its influence over reward circuitry via projections to the ventral tegmental area (45, 46). Furthermore, the cerebellum has been implicated in indirectly affecting reinforcement learning by reducing motor noise (47). Considering the cerebellum’s established role in reward learning, we hypothesize that it may regulate the extent to which dopaminergic prediction error signals in the NAc contribute to updating previously acquired value representations.

Regarding cost valuation, we demonstrated distinct functional neural correlates for cognitive effort discounting in the AIC, while those for temporal discounting were not observed. Previous studies indicate partially separate valuation networks for delay and effort costs, including the ventromedial prefrontal cortex, ventral striatum, posterior cingulate cortex, and lateral parietal cortex for delayed reward valuation, the AIC and anterior cingulate cortex (ACC) for effort valuation, and the right orbitofrontal cortex and lateral temporal and parietal cortices for encoding the value of chosen options related to delay and effort discounting (16, 48). The lack of observed neural correlates for temporal discounting may be due to the limitations of resting-state functional connectivity in capturing the temporal variability required for valuation. Task-activated imaging data might be more suitable for examining the neural correlates of temporal discounting.

Our model-based analysis revealed the role of the left AIC and aMCC network in modulating cognitive effort valuation, a finding that is partially consistent with prior studies. Previous research demonstrated the role of AIC and ACC in decision-making based on effortful reward, distinct from effects of reward delays (16, 49). While prior studies primarily investigated physical effort, Aben et al. (50) established the dorsal ACC’s involvement in processing cognitive effort demands through its connectivity with task-specific cortical regions. The adjacent aMCC is also functionally connected to the AIC, as demonstrated during both resting and task performance, and is associated with cognitive-motor control (51). Moreover, Touroutoglou et al. (52) have suggested the aMCC’s role in motivation by predicting energy needs and guiding behavior toward allostatic energy balance. We observed activity in the aMCC instead of more rostral ACC regions because our task primarily required motor execution and cognitive control and did not elicit conflict or incorporate decision-making. Our results expand on prior findings by linking AIC-aMCC connectivity to cognitive effort discounting and its impact on performance variability. This association reflects the implicit influence of effort valuation in cognitive processing.

Furthermore, our results showing the effects of effort discounting on incentive valence has been underexplored. Notably, Hernandez Lallement et al. (18) showed that cost-benefit valuation may engage distinct neural networks for gains and losses. They found that the AIC activity is significantly modulated by loss magnitude when effort is involved. Our observations of the AIC functional connectivity related to loss aversion and effort discounting are consistent with and extend their findings.

Notably, we observed laterality in the right and left anterior insular cortex (AIC) differentially associated with loss aversion and cognitive effort discounting, respectively. Lateralized engagement of the AIC has been observed in different aspects of loss aversions in prior studies. Specifically, a right dominance was observed for aversive salience (53, 54), while bilateral or left-lateralized activation was found in learning-related risk prediction errors (55–57). Therefore, our observed association between resting-state functional connectivity in the right AIC and loss aversion aligns with the AIC’s role in saliency processing.is congruent with its role in the salience component of loss aversion.

Conversely, the left lateralization of the AIC in cognitive discounting observed in our study contrasts with some prior studies reporting right AIC activation related to expected effort cost, effort prediction during probabilistic learning, and the valuation of prospective effort (58, 59). However, this finding may reflect a resting-state network involved in the subjective experience of task demand, consistent with prior studies reporting left AIC activation during self-evaluation of mental effort investment modulated by task demands (60).

Crucially, we were able to demonstrate the relationship between baseline AIC-aMCC connectivity and amotivaton-related tendency to discount cognitive effort during a working memory task using model-based analysis. These results implicate the AIC-aMCC network as a potential pathophysiological mechanism underlying concentration difficulties in depression. Our findings therefore imply that enhancing baseline salience network function, particularly concerning cognitive effort valuation, could be a promising therapeutic target for depressive disorders.

A key limitation of this study is the overall small sample size. Given the number of parameters included in our computational and regression models, small sample size could decrease the statistical power of our analyses and increase the potential for overfitting, which might affect the generalizability of our findings. Another limitation is the absence of task-based fMRI data to match and confirm that the functional connectivities demonstrated by model-based analyses are the brain regional network recruited during task performance. Lastly, the inclusion of nine medium-risk drinkers in our sample could introduce confounding effects on the behavioral and neural results. Prior studies report that severe alcohol use can diminish neural capacity in working memory task performance and affect resting-state neural networks (61, 62). However, we did not observe any correlations between AUDIT scores and either task accuracy rates or resting-state functional connectivity within our sample.

In conclusion, we found that among components of cost-benefit analysis in cognitive performance, devaluation of effort, involving the AIC-aMCC network, may underly a general mechanism of amotivation. We demonstrated that a computational model of temporal difference learning and value discounting is feasible for examining the implicit behavioral effect of cost-benefit analysis and identifying the subconstruct behind amotivation. Furthermore, our results suggest that interventions targeting the salience network to modulate effort valuation as a potential therapy for treating amotivation. To validate the applicability of this model and to establish the pathophysiological role of these findings in depressive disorders, larger-scale studies are required for cross-validation and comparison between individuals with major depressive disorder and healthy controls.

## Data Availability

The raw data supporting the conclusions of this article will be made available by the authors, without undue reservation.
