# Supplementary material for: Cognitive effort devaluation and the salience network: a computational model of amotivation in depression
Source: Front Psychiatry. 2025 Sep 1;16:1581802. doi: 10.3389/fpsyt.2025.1581802 (PMC12434043; doi:10.3389/fpsyt.2025.1581802)
Supplement: Supplementary file 1 [file Table1.docx]

| **Supplementary Table 1**. Model comparison in individual subjects | | | | | |
| --- | --- | --- | --- | --- | --- |
| Subject | Depression | Responsibility | | Log evidence | |
|  |  | 2-TD | 2-LR | 2-TD | 2-LR |
| MFCE002 | N | 1 | 1.24E-78 | -251.90 | -254.55 |
| MFCE004 | N | 1 | 1.40E-06 | -287.81 | -289.11 |
| MFCE005 | D | 1 | 3.98E-18 | -293.03 | -294.89 |
| MFCE006 | D | 1 | 2.74E-07 | -319.16 | -317.08 |
| MFCE008 | D | 1 | 2.50E-52 | -280.70 | -283.49 |
| MFCE009 | D | 1 | 1.07E-14 | -290.98 | -293.42 |
| MFCE010 | N | 1 | 4.34E-20 | -257.02 | -257.50 |
| MFCE011 | N | 1 | 2.77E-24 | -285.68 | -286.50 |
| MFCE012 | D | 0.99641 | 0.00359 | -273.65 | -273.16 |
| MFCE013 | N | 1 | 4.08E-20 | -286.61 | -288.09 |
| MFCE014 | N | 1 | 5.10E-15 | -243.56 | -241.50 |
| MFCE015 | N | 1 | 1.73E-22 | -280.05 | -281.16 |
| MFCE016 | D | 1 | 1.80E-13 | -316.76 | -317.96 |
| MFCE017 | N | 1 | 1.54E-23 | -167.69 | -170.79 |
| MFCE018 | N | 1 | 1.15E-14 | -291.54 | -293.23 |
| MFCE032 | N | 1 | 2.00E-11 | -315.31 | -315.90 |
| MFCE035 | N | 1 | 1.06E-16 | -327.50 | -329.51 |
| MFCE037 | N | 1 | 4.98E-35 | -187.78 | -189.97 |
| MFCE041 | N | 1 | 8.84E-38 | -195.02 | -196.42 |
| MFCE042 | N | 1 | 1.38E-13 | -135.55 | -137.87 |
| MFCE043 | D | 1 | 1.05E-39 | -197.16 | -199.60 |
| MFCE044 | N | 1 | 1.13E-20 | -267.97 | -270.02 |
| MFCE045 | N | 0.99999 | 1.10E-05 | -312.18 | -312.64 |
| MFCE046 | N | 1 | 3.55E-16 | -305.64 | -308.08 |
| MFCE049 | D | 1 | 2.60E-16 | -320.95 | -321.77 |
| MFCE053 | N | 1 | 1.79E-08 | -285.52 | -286.90 |
| MFCE060 | N | 1 | 4.31E-22 | -213.32 | -215.23 |
| MFCE061 | N | 1 | 1.10E-17 | -282.74 | -286.16 |
| MFCE062 | N | 1 | 8.22E-26 | -243.79 | -247.50 |
| MFCE067 | N | 1 | 5.34E-19 | -97.07 | -91.40 |
| MFCE072 | N | 1 | 8.06E-10 | -299.31 | -300.33 |
| MFCE073 | N | 2.04E-05 | 0.99998 | -285.79 | -271.84 |
| MFCE076 | D | 1 | 1.04E-21 | -356.27 | -351.97 |
| MFCE082 | N | 1 | 6.25E-08 | -398.65 | -400.37 |
| MFCE086 | N | 1 | 1.81E-12 | -312.78 | -313.90 |
| MFCE091 | D | 1 | 2.62E-17 | -355.60 | -357.50 |
| MFCE092 | D | 1 | 6.37E-21 | -327.14 | -330.32 |
| MFCE099 | D | 1 | 1.63E-20 | -295.73 | -297.62 |
| MFCE100 | N | 1 | 2.77E-33 | -147.99 | -150.75 |
| MFCE101 | N | 1 | 1.78E-10 | -297.09 | -294.78 |
| MFCE102 | N | 1 | 1.83E-33 | -145.60 | -148.14 |
| MFCE105 | N | 1 | 9.49E-32 | -62.76 | -65.14 |
| MFCE106 | N | 1 | 1.60E-19 | -200.70 | -202.43 |
| 2-TD, two-temporal discount model; 2-LR, two-learning rate model;  N, no/minimum depression; D, mild to severe depression  Mild to severe depression (D) was defined as Beck Depression Inventory score greater than 13. | | | | | |
