# Supplementary material for: Cognitive effort devaluation and the salience network: a computational model of amotivation in depression
Source: Front Psychiatry. 2025 Sep 1;16:1581802. doi: 10.3389/fpsyt.2025.1581802 (PMC12434043; doi:10.3389/fpsyt.2025.1581802)
Supplement: Supplementary file 2 [file Table2.docx]

| **Supplementary Table 2**. The parameter estimates from the two-temporal discount model | | |
| --- | --- | --- |
| Parameters | All Subjects  (n=43) | No/Minimum Depression  (n=31) |
| *Beta (cognitive capacity)* | 4.884 (1.503) | 5.014 (1.385) |
| *Lambda (loss aversion)* | 1.108 (0.217) | 1.108 (0.207) |
| *Gamma (cognitive effort discount)* | 0.505 (0.399) | 0.457 (0.381) |
| *kappa1 (PR temporal discount)* | 0.899 (0.145) | 0.899 (0.161) |
| *kappa2 (NR temporal discount)* | 0.785 (0.224) | 0.785 (0.155) |
| *Alpha (learning rate)* | 0.023 (0.031) | 0.025 (0.038) |
| No/Minimum Depression defined by Beck Depression Inventory score range of 0~13.  PR, positive reinforcement; NR, negative reinforcement.  The median (interquartile range) is presented. | | |
