# Supplementary material for: Cognitive effort devaluation and the salience network: a computational model of amotivation in depression
Source: Front Psychiatry. 2025 Sep 1;16:1581802. doi: 10.3389/fpsyt.2025.1581802 (PMC12434043; doi:10.3389/fpsyt.2025.1581802)
Supplement: Supplementary file 4 [file DataSheet2.docx]

**Supplementary Methods S2.** Code for the two-learning rate model. The MATLAB code is based on the Hierarchical Bayesian Inference codes by Piray et al. (2019)

%%% Effort-based reinforcement model: 2-LR

% -Loss aversion model: reward size, type effect

% -Hyperbolic discount model: cognitive effort discount

% -Temporal difference learning

% : Separate PR & NR learning rate

function [loglik] = Model_CEBRZ4a(parameters,subj)

%% parameters(6)

nd_beta = parameters(1); % cognitive capacity factor

beta = exp(nd_beta);

nd_lambda = parameters(2); % loss aversion factor

lambda = exp(nd_lambda);

nd_gamma = parameters(3); % cognitive effort discount factor

gamma = 1/(1+exp(-nd_gamma)); % range 0~1

nd_kappa = parameters(4); % temporal discount rate

kappa = 1/(1+exp(-nd_kappa)); % range 0~1

nd_alpha1 = parameters(5); % PR learning rate

alpha1 = 1/(1+exp(-nd_alpha1)); % range 0~1

nd_alpha2 = parameters(6); % NR learning rate

alpha2 = 1/(1+exp(-nd_alpha2)); % range 0~1

%% set initial expected cue value

qv0 = 1;

%% unpack data

block = subj.block; % block 1. 0-back, 2. 2-back

cue = subj.cue; % cue number(1~10: PN,P1L,P2L,P1H,P2H,NN,N1L,N2L,N1H,N2H)

outcome = subj.outcome; % response outcome(1 correct, 0 incorrect)

reward = subj.reward; % reward received (0, 1, 2)

cload = subj.nback; % n-back load/cognitive effort level (0, 2)

rtype = subj.rwtype; % reward type (0 PR, 1 NR)

%% set variable values

nt = size(outcome,1); % number of trials

[~,b] = unique(block);

%% set cue value array

% array columns: PN, P1L, P2L, P1H, P2H, NN, N1L, N2L, N1H, N2H

QV = qv0*ones(1,10); % initial expected cue value

%% set data arrays for storing computed probabilities & values

p = nan(nt,1); % probability of correct response

uv = nan(nt,10); % updated cue values

for n=1:nt

%% new input parameters

q = cue(n); % cue

o = outcome(n); % response outcome (1 correct, 0 incorrect)

r = reward(n); % reward received/loss avoided (0,1,2)

c = cload(n); % n-back load (cognitive effort level)

t = rtype(n); % reward type (P,p: 0, N,n: 1)

%% reset cue value for n-back block change

if n == b(2)

QV = qv0*ones(1,10); % initial expected cue value = 1000 Korean won

end

%% set cue value

v = QV(q);

%% Effort-based reinforcement value function

% Reward type model

% - if x>=0, u = x^rho; if x<0, u = -lambda*(-x)^rho < 0

% - loss aversion factor (lambda)

% - degree of risk aversion (rho: 1 neutral, <1 risk-aversive, >1 risk-seeking)

% - set rho=1; u>0; t=0 for PR, 1 for NR

% Cognitive effort discount model

% - hyperbolic function, u = x/(1+gamma*c)

U = lambda^t*v/(1+gamma*c);

%% cue value learning and update

% temporal difference learning

QV(q) = v + ((1-t)*alpha1+t*alpha2) * (r + kappa*U - v);

%% probability of correct response

% sigmoid function transformation to 0~1 value

% accuracy = beta*U

% if beta=0, Pcr=1/4 (random response from 4 choices)

Pcr = 1/(1+3*exp(-beta*U));

%% store probability of correct response

if o==1; p(n) = Pcr; % accuracy rate

elseif o==0; p(n) = 1 - Pcr; % error rate

end

%% store updated cue values

uv(n,q) = QV(q);

end

%% log-likelihood is defined as the sum of log-probability of choice data

% (given the parameters).

loglik = sum(log(p+eps));

% Note that eps is a very small number in matlab,

% which does not have any effect in practice,

% but it overcomes the problem of underflow when p is very very small

% (effectively 0).

end
